# Exercise Self-Efficacy and patient global assessment were associated with 6-min walk test distance in persons with rheumatoid arthritis

**DOI:** 10.1007/s10067-022-06309-6

**Published:** 2022-08-05

**Authors:** Ingrid Sæther Houge, Mari Hoff, Oddrun Halsan, Vibeke Videm

**Affiliations:** 1 Department of Clinical and Molecular Medicine, NTNU – Norwegian University of Science and Technology, St. Olavs University Hospital, Lab Center 3 East, NO-7006 Trondheim, Norway; 2 Department of Neuromedicine and Movement Science, NTNU – Norwegian University of Science and Technology, Trondheim, Norway; 3 Department of Rheumatology, St. Olavs University Hospital, Trondheim, Norway; 4 Department of Rheumatology, Levanger Hospital, Levanger, Norway; 5 Department of Immunology and Transfusion Medicine, St. Olavs University Hospital, Trondheim, Norway

**Keywords:** “6-min walk test, “Functional capacity, “Patient-reported outcome measures, “Rheumatoid arthritis

## Abstract

**Introduction:**

Low functional capacity is related to future loss of daily function and cardiovascular events. The present study explored the associations of patient-reported outcome measures (PROMs) and disease-specific measures with functional capacity as measured by the 6-min walk test (6MWT) in persons with rheumatoid arthritis (RA).

**Methods:**

Seventy-nine participants from rheumatology outpatient clinics were included. The distance walked during the 6MWT (6MWD) was the dependent variable in multivariable regression analyses. Model 1 included the independent variables sex, age (in tertiles to improve model fit), and body mass index (BMI). Building on Model 1, Model 2 added smoking, patient global assessment (PGA), Exercise Self-Efficacy, Hospital Anxiety and Depression Scale’s Depression score, and Cohen’s Perceived Stress Scale score, whereas Model 3 added smoking, disease duration, present use of glucocorticosteroids, seropositivity, Disease Activity Score 28—C-Reactive Protein (DAS28-CRP), and a comorbidity variable.

**Results:**

Median age was 65 years, 76% were female, and median 6MWD was 493 m. In Model 1, BMI and age were significantly associated with the 6MWD (*R*^2^ = 0.42). In Model 2, PGA and Exercise Self-Efficacy were also significantly associated with the 6MWD, with standardized regression coefficients of − 0.21 (*p* = 0.03) and 0.26 (*p* = 0.004) respectively (*R*^2^ = 0.54). The RA-specific variables in Model 3 were not significantly associated with the 6MWD (*R*^2^ = 0.49).

**Conclusion:**

The PROMs PGA and Exercise Self-Efficacy were significantly associated with functional capacity as measured by the 6MWT in persons with RA, whereas disease-specific measures such as DAS28-CRP and disease duration were not.

**Table:** 

**Key Points** *• Functional capacity measured with the 6-minute walk test was significantly associated with body mass index, age, patient global assessment, and Exercise Self-Efficacy in persons with RA.* *• Patient-reported outcome measures explained more of the variation in functional capacity than objective or composite measures of disease and are relevant measures in clinical follow-up.* *• Techniques that enhance self-efficacy for exercise should be incorporated into clinical practice to promote physical activity.*	

## Introduction

Rheumatoid arthritis (RA) is a systemic inflammatory joint disease, affecting 0.5–1.0% of the adult population, and is associated with depression, hypertension, cardiovascular disease (CVD), pulmonary disease, and increased mortality rates [1–3]. Low cardiorespiratory fitness (CRF) is an important mediator of the excess mortality among persons with RA, and increasing the physical activity (PA) and fitness levels in this group is a central part of CVD risk management [4]. The European Alliance of Associations for Rheumatology (EULAR) recommends that PA and exercise should be an integral part of standard care for persons with RA [5]. Healthcare professionals should promote PA, refer to interventions if necessary, and emphasize to the patients that the PA recommendations for the general population are safe and applicable for persons with RA [5–7].

Compared to the general population, fewer persons with RA meet the PA recommendations [8, 9]. Interventions targeting persons with low PA level and low CRF are therefore needed. Cardiopulmonary exercise testing is the gold standard method for assessing CRF. However, as testing requires personnel and special equipment, and has several contra-indications, it is not practical in clinical settings. Non-exercise formula may be applied to estimate CRF (eCRF), which is a quick method that does not depend on a physical test [10, 11]. The 6-min walk test (6MWT) is another method to assess overall functional capacity with an objective test, in which the distance walked in 6 min (6MWD) is registered [12]. The 6MWT is safe to perform, requires few resources, and gives information relevant to daily function. A recent large study found that the 6MWD could predict very low CRF in the general population, thereby identify persons in which interventions are most strongly needed [13].

Patient-reported outcome measures (PROMs) are calculated from questionnaires which the patients complete. Such measures capture the impact of disease in different and complementary manners compared to the objective measures of disease. The inclusion of PROMs in standard care reflects the focus on patient-centered follow-up. A vast number of PROMs exist in rheumatology, related to disease activity, function, psychological aspects etc. One of the most widely used is the patient global assessment (PGA), in which the patients rate their disease activity and indirectly the burden of RA, which may include consideration of symptoms, joint damage, function, disease activity, as well as psychological and societal aspects [14]. In a study testing CRF in persons with RA, the PGA was associated with CRF, whereas other RA-specific measures such as physician global assessment and swollen joint counts were not [15]. Exercise Self-Efficacy is a PROM assessing the psychological concept of self-efficacy for exercise; the belief in one’s ability to perform exercise under different circumstances [16]. Previous studies have found that Exercise Self-Efficacy correlated with actual PA behaviour and the ability to change behaviour, and that interventions could increase self-efficacy [17–20].

The hypotheses for the present study were that PROMs would have a stronger association with functional capacity measured by the 6MWT than objective or composite disease measures, and that the eCRF would be significantly associated with the 6MWD in persons with RA. The primary aim was to investigate the association between several different factors and the 6MWD in persons with RA. The secondary aim was to explore the relationship between the eCRF and the 6MWD in persons with RA.

## Participants and methods

### Participants

The present investigation was part of a larger study regarding PROMs and PA in patients with inflammatory arthritis, FysKond2. Participants fulfilling the American College of Rheumatology/EULAR 2010 criteria for RA were recruited from the Rheumatology outpatient clinics at Levanger Hospital and St. Olavs University Hospital in Norway in 2019 and 2021 [21]. The participants received an information letter before an appointment and were approached about the project when they came to the hospital. The participants were asked to perform the 6MWT, which was an optional part of their participation.

### Questionnaires and measurements

The participants filled in previously published questionnaires (further described below) related to psychological and disease-specific PROMs. They also responded to questions regarding PA and background information. Resting heart rate was measured after the participants had been sitting for at least 5 min.

The Exercise Self-Efficacy score is based on 5 questions regarding ability to perform regular PA under different circumstances; when tired, when in a bad mood, when there is lack of time, during holidays, and when it rains/snows [16]. Each question was rated on a Likert scale from 1 to 7, where 1 was anchored as “completely disagree” and 7 as “completely agree”. The scores for each question were added, giving an overall possible range of 5 to 35. Higher scores indicate higher self-efficacy for exercise.

Presence of depressive symptoms in the past week was measured with the 7 questions in the Hospital Anxiety and Depression Scale’s Depression score (HADS-D). HADS-D ranges from 0 to 21; higher scores imply more depressive symptoms [22].

Level of stress in the past month was assessed with Cohen’s scale for perceived stress, which is based on 10 questions [23]. The score ranges from 0 to 40; higher scores indicate more stress.

The PGA where the participants rated overall disease activity on a 100-mm visual analogue scale was assessed using the following phrasing: “Please consider the activity of your rheumatic disease in the past week. When considering all the symptoms, how do you think your state is?” A higher score implies more symptoms.

The modified Stanford Health Assessment Questionnaire (mHAQ) was used to measure self-reported physical function in the past week [24]. The mHAQ score is calculated from 8 questions, the final score ranges from 0 to 3, and a lower score indicates better physical function.

Information about duration, frequency, and intensity of the participants’ habitual PA was used to evaluate whether they fulfilled the 2007 recommendations for PA from the American College of Sport Medicine and the American Heart Association (ACSM/AHA) [6]. The information about the participants’ habitual PA was also applied to calculate a PA index as previously described [11]. A non-exercise model specific for persons with RA was used to estimate CRF based on sex, body mass index (BMI), smoking habit, PGA, resting heart rate, and the PA index [10].

The 6MWT was performed using standardized instructions from the American Thoracic Society [12]. The participants walked back and forth along a 25-m stretch and were instructed to walk as far as possible for 6 min. The participants were allowed to use their normal walking aids, choose their walking pace, and could stop to rest if necessary. They assessed their level of perceived exertion before and after the test using a Borg scale from 6 (no exertion) to 20 (maximum exertion), which is a scale closely related to physiological measures of exercise intensity like heart rate [25, 26]. The participants also rated their level of dyspnoea and lower extremity pain on similar scales from 6 (none) to 20 (maximum) before and after the test. The heart rate was measured immediately after the test.

### Hospital records

A review of hospital records was performed to collect information regarding the diagnosis, use of disease modifying anti-rheumatic drugs (DMARDs) and glucocorticosteroids, comorbidities, seropositivity status, and disease activity. Seropositivity was defined as a positive test for rheumatoid factor and/or anti-citrullinated protein antibody. Disease activity was assessed using the last documented Disease Activity Score 28—C-Reactive Protein (DAS28-CRP). DAS28-CRP is a composite score incorporating both subjective and objective measures of disease activity, namely, swollen and tender joint counts, CRP, and PGA. For 19% of the participants, the last recorded DAS28-CRP value was from more than one year before inclusion to the study.

### Statistics

Normality of continuous variables was assessed visually and with the Shapiro–Wilk test. As most of the continuous variables were not normally distributed, continuous data are presented as median with 25^th^ and 75^th^ percentile and compared with the Mann–Whitney *U*-test. Categorical variables are presented as number with percentage and were compared with the Chi-square test. Statistical analyses were performed using Stata (v16, StataCorp). *p*-values < 0.05 were considered statistically significant.

The associations between the different variables and the 6MWD were analysed using multivariable linear regression, with the 6MWD as dependent variable. Model 1 included variables known to be associated with the 6MWD; sex, age, and BMI. Model 2 explored the additional associations of several PROMs while Model 3 focused on more objective and composite RA measures. Model 2 added smoking habit (dichotomized as never or ever smoker), Cohen’s scale of perceived stress, HADS-D, Exercise Self-Efficacy, and PGA to Model 1. Model 3 added smoking habit, duration of RA, DAS28-CRP, seropositivity, present use of glucocorticosteroids, and comorbidities to Model 1. The comorbidity variable was coded “yes” if the participants had a history of any of the following: hypertension, angina, myocardial infarction, arrythmia, stroke, chronic obstructive respiratory disease, chronic restrictive respiratory disease, asthma, diabetes, or cancer; or “no” if not.

Age was categorized into tertiles to improve model fit (< 59, 59–69, or ≥ 70 years). Participants with missing data for variables in the models were excluded. Residual plots, Akaike and Bayesian information criteria, and *R*^2^ were applied to evaluate model assumptions and model fit. Standardized regression coefficients, which is the number of standard deviations the dependent variable changes per standard deviation increase in each of the independent variables, were calculated to allow for direct comparison of the coefficients of the variables in the models.

A sensitivity analysis assessed whether recruitment at Levanger Hospital (2019) or at St. Olavs University Hospital (2021) had a significant impact on the 6MWD, as the COVID-19 pandemic started between the two data collection periods. A regression model was performed with the 6MWD as the dependent variable, and the independent variables sex, age (in tertiles), BMI, and hospital (Levanger or St. Olavs University Hospital).

In the secondary analysis, the relationship between the eCRF and the 6MWD was assessed with Pearson’s correlation coefficients and a scatterplot. Several variables like sex and age are part of the eCRF formula, and multivariable regression was not considered an appropriate analytical method due to collinearity issues.

### Ethics

The study was approved by the Regional Committee for Medical and Health Research Ethics (#23,420). All participants gave informed consent. The study was performed in accordance with the principles of the Helsinki declaration.

## Results

**Fig. 1:**
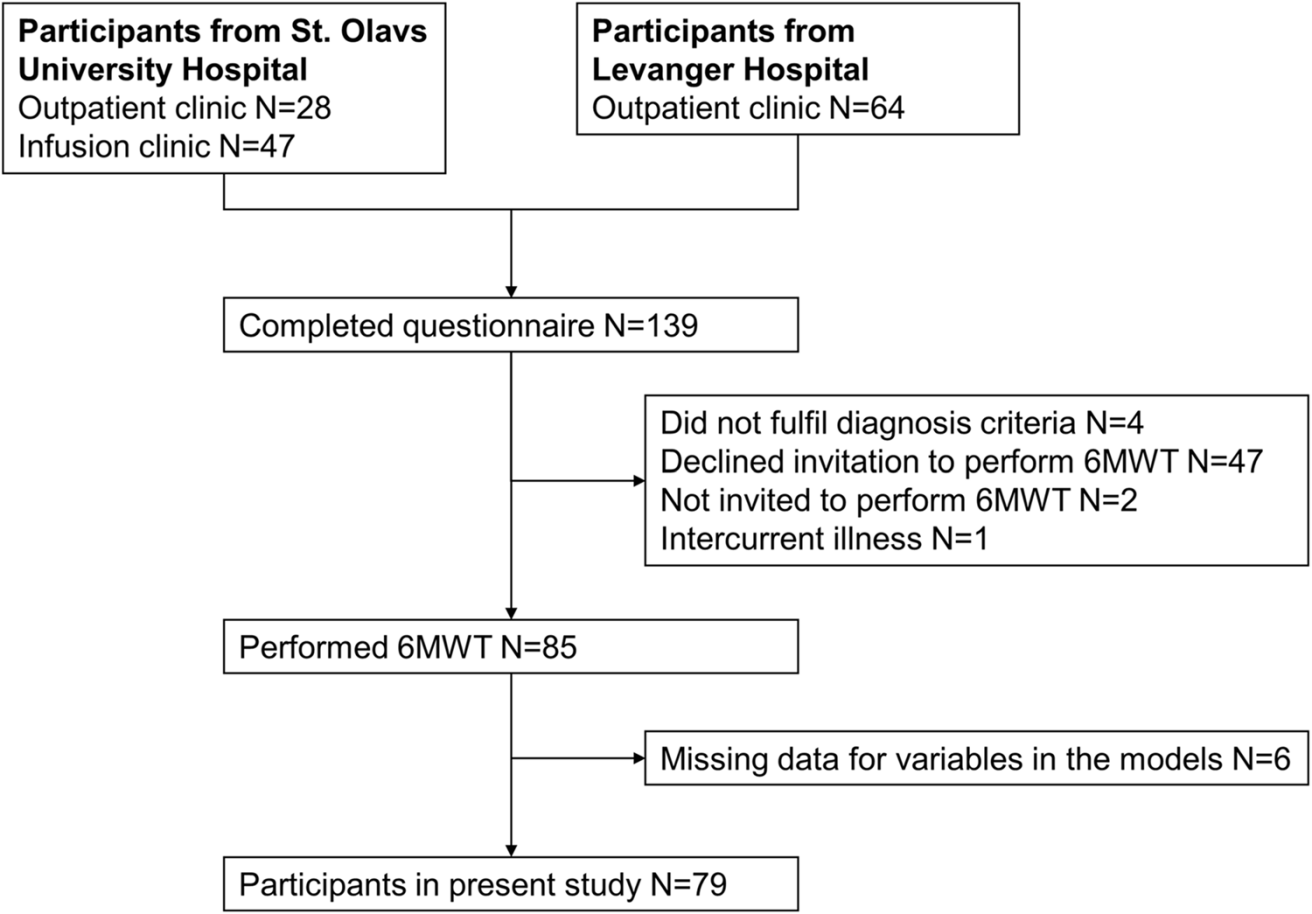
Inclusion and exclusions to the present study. Abbreviation: 6MWT: 6-min walk test. Two participants were not invited to perform the 6MWT for practical reasons. One participant was excluded due to an intercurrent disease that affected the result of the 6MWT

Out of the 200 persons that were invited to participate, 139 completed the questionnaires (70%) and 86 performed the 6MWT (43%). The participants in the overall study were somewhat younger than those who declined the invitation (median age 64 versus 67 years, *p* = 0.03), and the proportion of females was similar (71% versus 82%, *p* = 0.11). Inclusions and exclusions to the study are presented in Fig. 1. Table 1 presents participant characteristics for the RA patients invited to perform the 6MWT, comparing the participants included in the analysis (*N* = 79) with participants who either declined to perform the 6MWT or were excluded from analysis for other reasons (*N* = 54).

**Table 1:** Participant characteristics for those invited to perform the 6-min walk test ${}^{\mathrm{a},\mathrm{b},\mathrm{c},\mathrm{d}}$

	Included in analysis (*N* = 79)	Not included in analysis (*N* = 54)	*p*-value
Female sex	60 (76)	36 (67)	0.24
Age (years)	65 (55, 71)	62 (53, 71)	0.44
Smoking status			0.56
Current smoker Former smoker Never smoker	7 (9) 42 (53) 30 (38)	3 (6) 34 (63) 17 (31)	
Body mass index (kg × ${\mathrm{m}}^{-2}$)	26.3 (23.6, 28.9)	27.7 (23.7, 31.5)	0.09
Resting heart rate (beats per minute)	68 (63, 76)	71 (68, 80)	0.06
Rheumatoid arthritis duration (years)	10 (5, 23)	6 (2, 15)	0.03
Age when diagnosed (years)	48 (35, 58)	50 (39, 61)	0.26
Seropositive (ACPA and/or RF)	68 (86)	48 (89)	0.63
Medication, current use			
Conventional DMARDs Biological DMARDs Glucocorticosteroids	67 (85) 42 (53) 21 (27)	42 (78) 30 (56) 14 (26)	0.30 0.79 0.93
Comorbidity			
Hypertension Osteoporosis Respiratory disease ${}^{\mathrm{e}}$ Cardiovascular disease ${}^{\mathrm{f}}$ Diabetes Cancer	32 (41) 23 (29) 20 (25) 13 (16) 7 (9) 5 (6)	16 (30) 12 (22) 9 (17) 12 (22) 3 (6) 5 (9)	0.20 0.38 0.24 0.40 0.48 0.52
DAS28-CRP			0.19
Remission Low disease activity Moderate disease activity High disease activity	52 (66) 14 (18) 12 (15) 1 (1)	27 (50) 10 (18) 15 (28) 2 (4)	
Overall score DAS28-CRP	2.3 (1.7, 2.9)	2.7 (1.8, 3.6)	0.14
Modified Stanford Health Assessment Questionnaire	0.38 (0.13, 0.63)	0.25 (0.00, 0.75)	0.46
Patient global assessment (mm)	27 (12, 40)	30 (14, 56)	0.16
Hospital Anxiety Depression Scale’s Depression score	3 (1, 4)	3 (1, 6)	0.26
Cohen’s Perceived Stress Scale	15 (9, 19)	15 (11, 19)	0.51
Exercise Self-Efficacy	25 (17, 30)	22 (17, 25)	0.07
Fulfills ACSM/AHA 2007 recommendations	23 (32)	11 (20)	0.28
Estimated cardiorespiratory fitness (mL x ${\mathrm{kg}}^{-1}$ x ${\mathrm{min}}^{-1}$)	29.0 (22.3, 35.5)	26.6 (22.6, 35.8)	0.79

^a^Number with percentage or median with 25^th^ and 75^th^ percentiles

^b^*ACSM/AHA*, American College of Sport Medicine/American Heart Association; *ACPA*, anti-citrullinated protein antibody; *DAS28-CRP*, Disease Activity Score 28—C—Reactive Protein; *DMARDs*, disease-modifying antirheumatic drugs; *RF*, rheumatoid factor

^c^Chi-square test or Mann–Whitney *U*-test

^d^Missing data: < 5% for all variables, with the following exceptions: estimated cardiorespiratory fitness (17% missing for those not included in the analysis), heart rate (11% missing for those not included in the analysis), mHAQ and HADS-D (7% missing data in the group not included in the analysis)

^e^Respiratory disease: chronic obstructive pulmonary disease, chronic restrictive pulmonary disease, or asthma

^f^Cardiovascular disease: angina, myocardial infarction, arrhythmia, or stroke

The median distance walked during the 6MWT was 493 m (range 248–738 m). The median values for perceived exertion, dyspnoea, lower extremity pain, and heart rate increased during the test (Table 2).

**Table 2:** Results from 6-min walk test ${}^{\mathrm{a},\mathrm{b}}$

Measures	Included patients (*N* = 79)
Distance walked (m)	493 (447, 576)
Heart rate	
Before the 6MWT After the 6MWT	68 (63, 73) 92 (84, 104)
Level of perceived exertion ${}^{\mathrm{c}}$	
Before the 6MWT After the 6MWT	9 (6, 11) 11 (9, 12)
Level of dyspnea ${}^{\mathrm{c}}$	
Before the 6MWT After the 6MWT	6 (6, 9) 11 (9, 13)
Level of pain in lower extremities ${}^{\mathrm{c}}$	
Before the 6MWT After the 6MWT	8 (6, 11) 9 (7, 13)
Walking aids	3 participants used one crutch (4)

^a^Number with percentage or median with 25^th^ and 75^th^ percentiles

^b^Abbreviation: *6MWT*, 6-min walk test

^c^Rated on a Borg scale from 6 (nothing) to 20 (maximum)

Results from the multivariable regression models are presented in Table 3. In Model 1, age and BMI were significantly associated with the 6MWD, whereas sex was not (*R*^2^ = 0.42). Among the additional variables in Model 2, Exercise Self-Efficacy and PGA were significantly associated with the 6MWD (*R*^2^ = 0.54). None of the additional variables in Model 3 was significantly associated with the 6MWD (*R*^2^ = 0.49). Compared to Model 1, Models 2 and 3 further explained 12% and 7% of the variation in the 6MWD, respectively.

**Table 3:** Variables associated with the 6-min walk test distance in multivariable regression analyses ${}^{\mathrm{a}}$

	Regression coefficient (95% CI)	Standardized regression coefficient	*p*-value
Model 1 (*R*^2^ = 0.42)			
Male sex	27 (− 9, 64)	0.13	0.14
Age			
Middle versus youngest tertile Oldest versus youngest tertile	− 35 (− 72, 1) − 115 (− 155, − 76)	− 0.20 − 0.59	0.06 < 0.001
Body mass index	− 8 (− 12, − 5)	− 0.39	< 0.001
Model 2 (*R*^2^ = 0.54)			
Male sex	15 (− 22, 53)	0.08	0.42
Age			
Middle versus youngest tertile Oldest versus youngest tertile	− 39 (− 74, − 3) − 117 (− 155, − 79)	− 0.22 − 0.60	0.03 < 0.001
Body mass index	− 6 (− 10, − 2)	− 0.28	0.002
Exercise Self-Efficacy	3 (1, 4)	0.26	0.004
Patient global assessment	− 1 (− 2, 0)	− 0.21	0.03
Cohen’s scale of perceived stress	− 2 (− 5, 1)	− 0.15	0.25
HADS-D	3 (− 4, 9)	0.11	0.38
Ever smoker	− 6 (− 39, 26)	− 0.03	0.71
Model 3 (*R*^2^ = 0.49)			
Male sex	28 (− 9, 66)	0.14	0.13
Age			
Middle versus youngest tertile Oldest versus youngest tertile	− 26 (− 64, 12) − 97 (− 142, − 53)	− 0.15 − 0.50	0.17 < 0.001
Body mass index	− 7 (− 11, − 3)	− 0.32	0.002
Ever smoker	− 27 (− 60, 7)	− 0.15	0.12
Comorbidity (yes/no)${}^{\mathrm{b}}$	− 23 (− 60, 15)	− 0.13	0.23
DAS28-CRP	− 12 (− 29, 5)	− 0.13	0.17
Present glucocorticosteroid use	− 24 (− 61, 12)	− 0.12	0.19
Seropositive (positive RF and/or ACPA)	8 (− 38, 55)	0.03	0.72
Rheumatoid arthritis duration	0 (− 1, 1)	− 0.01	0.94

^a^Abbreviations: *ACPA*, anti-citrullinated protein antibody; *CI*, confidence interval; *DAS28-CRP*, Disease Activity Score 28—C—Reactive Protein; *DMARD*, disease-modifying antirheumatic drugs; *HADS-D*, Hospital Anxiety and Depression Scale’s Depression score; *RF*, rheumatoid factor

^b^Comorbidity defined as having a history of hypertension, angina, myocardial infarction, arrythmia, stroke, chronic obstructive respiratory disease, chronic restrictive respiratory disease, asthma, diabetes, or cancer

In the Sensitivity analysis there was no significant association between which hospital the participants were recruited from and the 6MWD (*p* = 0.39), indicating that whether they participated in 2019 or 2021 did not have a major effect on the distance walked.

**Fig. 2:**
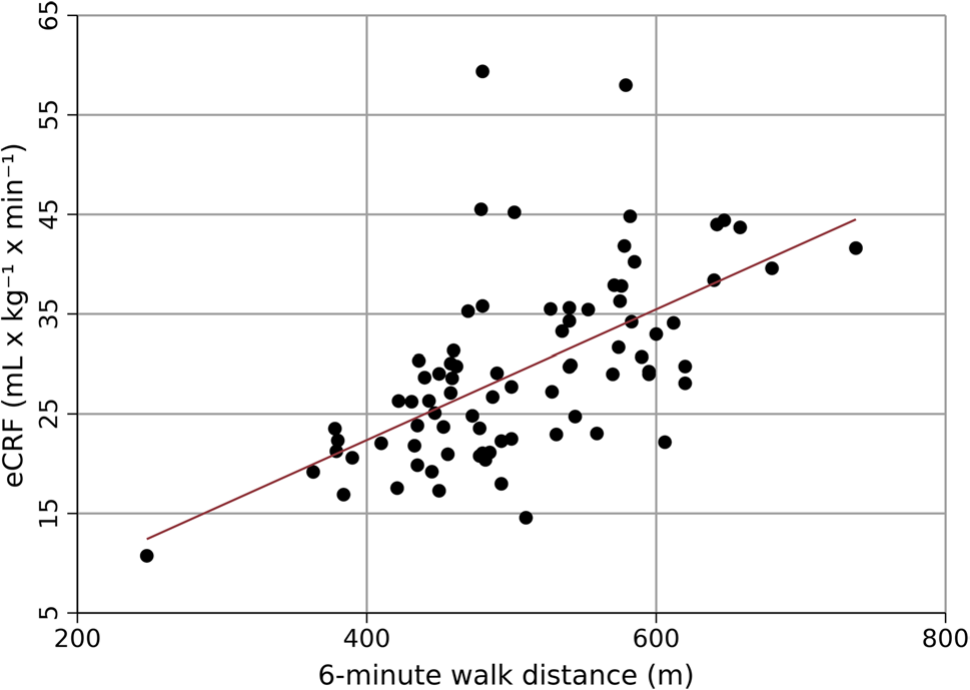
Estimated cardiorespiratory fitness versus 6-min walk distance. Abbreviation: eCRF: estimated cardiorespiratory fitness. Linear regression line presented. Note that the X and Y axes do not start at 0

The secondary analysis demonstrated that the eCRF was significantly associated with the 6MWD among persons with RA (Fig. 2). Pearson’s correlation coefficient was 0.61 (*p* < 0.0001). Three persons were excluded before the secondary analysis because of missing data for resting heart rate, which is needed to calculate eCRF.

## Discussion

The main finding of the present study was that Exercise Self-Efficacy and PGA, in addition to age and BMI, were significantly associated with functional capacity in persons with RA. Sex, smoking, level of depressive symptoms, level of perceived stress, seropositivity, disease duration, DAS28-CRP, use of glucocorticosteroids, and comorbidities had little impact on the distance walked. The eCRF was significantly associated with the 6MWD. The results confirmed the hypothesis that PROMs explained functional capacity better than more objective or composite measures of disease in this sample of RA patients.

The included participants had relatively high self-reported function and low disease activity levels, comparable to previous results from Norwegian RA patients on stable treatment [15, 27]. The median 6MWD for our patients was 493 m, which was relatively long compared to results from persons with other chronic diseases [28, 29]. The mean 6MWD in a study in heart failure patients was 222 m and 73% of the participants walked less than 300 m [28]. The mean 6MWD in cardiac surgery patients admitted to rehabilitation was 248 m, which increased to 374 m before discharge [29]. Among persons with RA, the initial mean 6MWD in two different studies were 391 m and 549 m, shorter and longer than the results in the present study, respectively [30, 31]. This suggests that the results from the 6MWT were in the expected range for persons with RA.

### Importance of PROMs

Exercise Self-Efficacy was significantly positively associated with the 6MWD in persons with RA, in line with results from other fields [32]. Interestingly, participants who performed the 6MWT had a trend towards higher Exercise Self-Efficacy compared to those who declined, indicating stronger beliefs in their ability to perform the test. Increasing self-efficacy may be a necessary step to turn intentions to increase PA into actual change of behaviour and thereby improvement of functional capacity. Examples of approaches that may increase self-efficacy are motivational interviewing techniques, self-regulation sessions, learning by doing, role modelling, positive feedback, problem solving, goal-setting, and education on body responses [19, 20]. To be able to engage in PA, persons with low self-efficacy for exercise may need supervision, more positive feedback and assurance, the option to exercise with peers, and an individualized exercise program, whereas persons with high self-efficacy may be less dependent on external factors [20].

The PGA is a subjective measure of disease activity that has been associated with CRF, and the present study found a significant negative association with the 6MWD [15]. The PGA is more closely related to the subjective impact of RA than to objective measures such as swollen joint count and inflammation [14, 33]. Furthermore, disease activity may affect which factors the PGA reflects. For example, health distress may only surface at lower disease activity [34]. As RA was relatively well controlled in most of our participants, their assessment may reflect a wider range of factors than pain and self-reported function. Several factors may affect both the PGA and PA behaviour, and some patient-reported barriers to PA including pain and fatigue may be improved by PA [14, 34–36].

Depressive symptoms may lead to a reduction in PA and thereby loss of functional capacity. However, our study did not find a significant association between depressive symptoms and the 6MWD in persons with RA. This is similar to findings among breast cancer survivors, but in contrast to results in heart failure patients [28, 37]. Potential explanations may be differences in patient characteristics, such as age, physical impairment, and level of depression. Compared to our participants, the heart failure patients were older, had higher scores for HADS-D (mean score 7), and walked shorter (mean 6MWD 222 m), whereas the breast cancer survivors were younger, had slightly higher HADS-D scores (mean score 5), and walked similar distances (mean 6MWD 511 m).

Perceived stress was not associated with the 6MWD in the present study. This was surprising as perceived stress has been negatively associated with PA in the general population and CRF in persons with diabetes [38, 39]. Maybe the association with stress would have been stronger in a sample with less variation in age. Participants with small children at home and a full-time job might experience more stress than a person who recently retired, but still walk further. Stress might interact with other psychological domains such as self-efficacy, and perhaps, the impact of stress was mediated through other variables in the model.

### Objective and composite disease measures

There were no significant associations between the 6MWD and the objective or composite RA measures. One study reported a significant negative association between DAS28 and the 6MWD (Spearman’s correlation coefficient − 0.294, *p* = 0.006); however, this was not further explored in multivariable analyses [30]. Perhaps the association between objective measures and the 6MWD would have been stronger in a population with higher disease activity or lower self-reported function.

### Clinical implications

Busy clinicians may prioritize objective over subjective measures of disease activity. Nevertheless, the fact that the objective and composite disease measures were not related to functional capacity underlines the importance of including PROMs in clinical evaluations. PGA is often used in the follow-up of persons with RA to represent the patient’s perspective regarding disease impact, whereas scoring of Exercise Self-Efficacy is not a common tool in rheumatology. Exercise Self-Efficacy assesses a psychological dimension that clinicians can target. One may include motivational interviewing techniques, goal-setting, and positive feedback as part of regular follow-up, or refer to exercise programs, physiotherapist etc. Interventions that target self-efficacy are probably most efficient when combined with some sort of exercise intervention [19]. In addition to prescribing medications, active promotion of PA and exercise is important as that is something the patients can do themselves to reduce symptoms and improve function [5, 7, 36].

### Functional capacity

The 6MWD was significantly associated with the eCRF in our study, similar to previous results showing moderate to high correlation with measured CRF in the general population and in persons with heart failure [13, 40]. The 6MWD is an objective measure of physical capacity that has been associated with important clinical outcomes like mortality and major cardiac events in other patient groups [29, 41, 42]. Furthermore, we have now shown that it is related to PGA, Exercise Self-Efficacy, and eCRF. The 6MWT is most often used to assess the effect of interventions, for example physical rehabilitation, exercise programs, or surgical procedures, but can also be part of initial clinical assessments. The testing itself may lead to conversations on aspects the patients perceive as important. A 6MWT is not equivalent to a cardiopulmonary exercise test as it does not measure CRF or give detailed information of cardiac and pulmonary function. Regardless, the test is simple, safe, and inexpensive to perform, and can help identify persons needing additional follow-up.

Another alternative to estimate physical capacity is using non-exercise formulas for eCRF [10]. Advantages of the formula approach is that it is quick, requires few measurements, and can be completed by the patients themselves [43]. A limitation of non-exercise formulas, at least in the general population, is that they may classify a large proportion of participants into a wrong fitness group [44].

### Representativeness

As 60% of those invited were not included in the analysis, a concern is whether the results are applicable to other persons with RA. The participants who performed the 6MWT and were included in the analysis were representative for those agreeing to participate in the study, apart from slightly longer disease duration. The persons who agreed to participate in the project (with or without performing the 6MWT) were slightly younger than those who declined. Only a third of the participants included in the analysis fulfilled the ACSM/AHA 2007 recommendations for PA, which is comparable to other Scandinavian studies of persons with RA [8, 9]. The results from the present study may not be applicable to patients with high disease activity and/or who live outside of Scandinavia.

### Strengths and weaknesses

A strength of the present study is the wide range of variables recorded, permitting investigation of several factors known to influence functional capacity. The participants varied in age, sex, disease activity level, and disease duration. The RA diagnoses were validated using hospital records, and information regarding comorbidity and medications had high accuracy. To our knowledge, this is the first study to thoroughly explore the association between different PROMs and disease-specific measures with the 6MWD in persons with RA.

A weakness is that the DAS28-CRP value might not reflect the present disease activity level for all participants. Evaluation of causation is not possible due to the cross-sectional design. A larger sample size would have increased the power of the study. For practical reasons the walking course for the 6MWT was 25 m, shorter than the recommended 30 m, which possibly led to a shorter distance walked due to more turns [45]. However, it is unlikely that this had a major impact on the associations between the investigated variables and the 6MWD. Lastly, as weight and height were self-reported, BMI estimates may be imprecise.

### Conclusion

The PROMs Exercise Self-Efficacy and PGA were significantly associated with functional capacity measured with the 6MWT in persons with RA, whereas objective and composite disease measures were not. Applying techniques that may enhance Exercise Self-Efficacy could help patients increase their PA level and thereby improve functional capacity. The present study also demonstrated that the eCRF was significantly associated with the 6MWD. The 6MWT is a cheap, objective, and feasible test that can help identify patients who could benefit from more comprehensive follow-up.
